# “I’m used to doing it by myself”: exploring self-reliance in pregnancy

**DOI:** 10.1186/s12884-018-2022-8

**Published:** 2018-10-05

**Authors:** Blair C. McNamara, Abigail Cutler, Lisbet Lundsberg, Holly Powell Kennedy, Aileen Gariepy

**Affiliations:** 1 Yale School of Medicine, 333 Cedar Street, New Haven, CT 06510 USA; 2 Department of Obstetrics, Gynecology and Reproductive Sciences, Yale School of Medicine, New Haven, CT USA; 3 Department of Obstetrics, Gynecology and Reproductive Sciences, Yale School of Medicine, New Haven, CT USA; 4 Yale School of Nursing, West Haven, CT USA; 5 Department of Obstetrics, Gynecology and Reproductive Sciences, Yale School of Medicine, New Haven, CT USA

**Keywords:** United States, Pregnancy, Self-reliance, Decision making, Social support

## Abstract

**Background:**

Self-reliance (the need to rely on one’s own efforts and abilities) is cited as a potential coping strategy for decreased or absent social support during pregnancy. Little data exists on how women view self-reliance in pregnancy.

**Methods:**

We recruited women from urban, walk-in pregnancy testing clinics from June 2014–June 2015. Women aged 16 to 44 and at less than 24 weeks gestational age were eligible. Participants completed an enrollment survey and in-person, semi-structured interviews. We used framework analysis to identify key concepts and assess thematic relationships.

**Results:**

Eighty-four English-speaking women completed qualitative interviews. Participants averaged 26 years of age and 7 weeks estimated gestational age. Most identified as Black (54%) or Hispanic (20%), were unemployed or homemakers (52%), unmarried (92%), and had at least one child (67%). Most did not intend to get pregnant (61%) and planned to continue their pregnancy and parent (65%). We identified self-reliance as a prevalent concept that almost half (48%) of participants discussed in relationship to their pregnancy. Self-reliance in pregnancy consisted of several subthemes: 1) past experiences, 2) expectations of motherhood, 3) financial independence, 4) decision making, and 5) parenting.

**Conclusions:**

Self-reliance is an important aspect of women’s reproductive lives and is threaded through women’s past and current thoughts, feelings, experiences and decisions about pregnancy. Women’s belief in their own self-reliance as well as a recognition of the limits of self-reliance merits further research, especially as a potential strategy to cope with decreased or absent social support during pregnancy.

**Electronic supplementary material:**

The online version of this article (10.1186/s12884-018-2022-8) contains supplementary material, which is available to authorized users.

## Background

Social support is defined as the receipt of resources, information, or emotional care through personal relationships [1, 2]. Increased social support during pregnancy and the postpartum period has been associated with decreased psychological distress during pregnancy [1, 3], faster progression of labor, higher Apgar scores, higher birthweight [2, 4], and reduced depression among new mothers and women who have abortions [1, 5–7]. Similarly, decreased or absent social support and increased psychological distress are linked to a variety of negative mental and physical health outcomes for pregnant women [1, 8, 9], including low birth weight and preterm delivery [3, 10–13], and postpartum depressive symptoms [14, 15].

When social support is low or absent, some have posited that pregnant women use resilience, optimism, and self-reliance as coping strategies [9, 16–20]. Resilience, defined as an ability to ‘bounce back’ after adversity, may act as a protective factor against psychologic stress and decreased social support during pregnancy [9, 16]. Optimism – described as a prospective belief that even without social support, a woman will be able to succeed using her own assets and abilities – has been found to be associated with decreased postpartum depression among pregnant women with low social support [17, 18]. Self-reliance is a similar but distinct concept from resilience or optimism and conveys a dependence on personal resources and abilities as opposed to those of others [19, 20]. Women may employ these potential coping strategies at various points in their reproductive lives and these strategies may intersect and overlap. An optimistic attitude can be a component of self-reliance, and resilient women can also be distinctly self-reliant, or intentionally reliant on others. Few studies have specifically examined self-reliance during the perinatal period, and focused on narrow, non-U.S. populations. Self-reliance has been described as a positive coping strategy for life stress and lack of social support among pregnant HIV-positive women in sub-Saharan Africa [19] and for first-time parents’ experiencing home-based postnatal care in Sweden [20].

Given the lack of research evaluating self-reliance among pregnant women, we address the concept of self-reliance as described by a diverse urban cohort of women following confirmation of a new pregnancy. Women discussed experiences with self-reliance as it related to previous and current pregnancies, the expectation of motherhood, finances, decision-making about the pregnancy, and parenting experiences.

## Methods

**Fig. 1:**
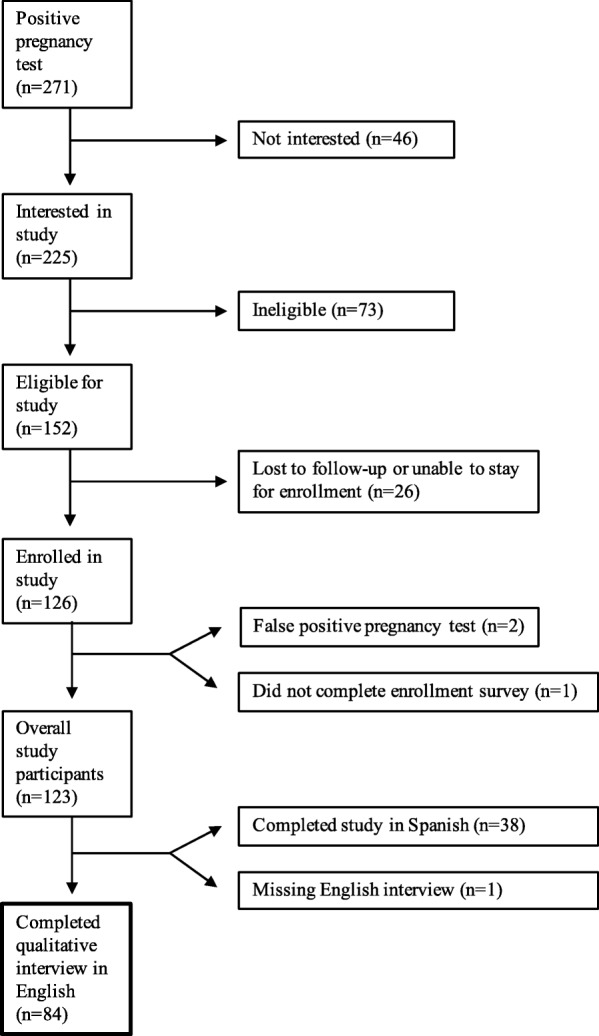
Participant recruitment and enrollment flow. A flow diagram depicting movement of participants from positive pregnancy test through to completed qualitative interview

We report on qualitative findings from a study conducted to explore the impact of a new pregnancy on women’s lives [21]. The overarching study recruited women presenting for pregnancy testing or abortion care at clinics in New Haven, CT, from June 2014 to June 2015. The data presented here were restricted to participants from pregnancy testing sites only, in order to focus on women with new pregnancy diagnoses who had not yet made a decision about how to resolve the pregnancy. Clinical staff referred interested women with positive pregnancy tests to the research team, who screened them for eligibility. Women were eligible if they were Spanish- or English-speaking, at a gestational age of < 24 completed weeks, 16–44 years old, and completed study enrollment within 1 week of their positive pregnancy test. Refer to Fig. 1 for a flow diagram of those participants screened, eligible, and enrolled in the study. Detailed study methods have been previously published [21]. In the state of Connecticut, pregnant women under the age of 18 are able to make all decisions regarding their pregnancy without parental input or consent. As such, our Institutional Review Board waived the need for parental consent for participants under the age of 18. Eighty-four participants completed in-depth qualitative interviews in English and are the basis of this analysis. Women who chose to participate in Spanish were analyzed separately and are not included in this investigation to ensure cross-language credibility [22].

All 84 participants completed an enrollment survey that collected demographic information (including age, race and ethnicity, relationship status, parity), measures of pregnancy intention, and plans for pregnancy termination or continuation. Enrolled participants were offered the opportunity to complete a one-on-one interview or a focus group interview (four women chose a group interview, which occurred as two two-person groups). Interviews were conducted by skilled research team interviewers using a semi-structured interview guide (Additional file 1: Figure S2) to ask participants open-ended questions about pregnancy intentions, initial and current thoughts and feelings after receiving a positive pregnancy test, and how they felt the pregnancy would impact their life, decisions, and relationships. All interviews were audio-recorded and transcribed, while maintaining confidentiality of the participants. We ascertained pregnancy outcome information (e.g. miscarriage, abortion, delivery) for each participant during a follow-up monitoring interview or through medical record review. We categorized pregnancy outcomes as miscarriage, abortion, or delivery. All participants provided written consent and received $50 cash as compensation for participation in the qualitative interviews. The study protocol was reviewed and approved by the Yale University Human Research Protection Program.

We used framework analysis to identify key concepts from our data and to assess thematic relationships [23]. We identified codes to evaluate common and dissimilar conceptual threads among interview transcripts. Four researchers (BM, AC, AG, LL) initially coded the same six interviews and then met to assess inter-coder reliability and generate a shared coding strategy and code list. Two independent coders (BM, AC) then coded the remaining transcripts and met regularly to assess discrepancies in coding. A senior methodologist and software expert (HPK), provided content-checking and guidance on all analysis. We then grouped codes thematically to draw conclusions about interactions and context in the interviews, and then re-evaluated the text using these themes. We used Atlas.ti (Berlin, Germany) to manage and code the transcripts.

## Results

At enrollment, participants averaged 26 years of age and 7 weeks estimated gestational age (EGA) (Table 1). Most identified as Black, non-Hispanic (54%) or Hispanic (20%). The majority reported less than or equal to a high school education (59%), were unemployed or homemakers (52%), were unmarried (92%), and had at least one child (67%). Some reported a previous history of depression (26%) or anxiety (25%). Previous miscarriage was reported by 40% and previous abortion was reported by 41%. When asked about the period just before becoming pregnant (pre-conception perspectives), 61% indicated they did not intend to get pregnant, 32% reported that they did not want to get pregnant and 25% indicated the pregnancy was not planned (Table 2). When asked about how they felt after learning they were pregnant, 29% reported that it was the wrong time to have a baby, 31% said the pregnancy was undesired, and only 13% said they were not happy with the pregnancy news (Table 2). At enrollment, 65% planned to parent, 18% planned abortion, 2% planned adoption, and 14% were unsure.

**Table 1:** Participant characteristics and sociodemographics, *N* = 84

Age, mean (SD)	26.1 (6.3)
Estimated gestational age at enrollment, weeks (SD)	7.2 (3.1)
Race-Ethnicity, *n* (%)	
Black, non-Hispanic	45 (54.2)
White, non-Hispanic	13 (15.7)
Hispanic	17 (20.5)
Multiracial, Other	8 (9.6)
Education, *n* (%)	
12 years/GED or less	49 (59.0)
Some college or college degree	34 (41.0)
Employment, *n* (%)	
Unemployed/homemaker	43 (51.8)
Full time/part time	40 (48.2)
Relationship status, *n* (%)	
Single, never married	42 (50.6)
Married	7 (8.4)
Living with partner, not married	19 (22.9)
Separated/divorced/widowed	15 (18.1)
Previous diagnosis of depression, *n* (%)	22 (26.2)
Previous diagnosis of anxiety, *n* (%)	21 (25.0)
Previous abortion, *n* (%)	34 (41.0)
Previous miscarriage, *n* (%)	32 (39.5)

**Table 2:** Measures of pregnancy context among participants, *N* = 84

Pre-conception perspectives	Intention, *n* (%)	
	Intended to get pregnant	17 (20.2)
	Intentions changing	16 (19.1)
	Did not intend to get pregnant	51 (60.7)
	Wanted, *n* (%)	
	Wanted to have a baby	23 (27.4)
	Mixed feelings	34 (40.5)
	Did not want to have a baby	27 (32.1)
	London measure of unplanned pregnancy, *n* (%)	
	Planned	17 (20.2)
	Ambivalent	46 (54.8)
	Unplanned	21 (25.0)
Post-conception perspectives	Timing, *n* (%)	
	Right time to have a baby	27 (32.1)
	Ok but not quite right	33 (39.3)
	Wrong time	24 (28.6)
	Desired pregnancy, *n* (%)	
	Yes	38 (45.2)
	No	26 (31.0)
	Not sure	20 (23.8)
	Happy about pregnancy, *n* (%)	
	Happy	54 (64.3)
	Neither happy/unhappy, not sure	19 (22.6)
	Unhappy	11 (13.1)
	Pregnancy plans, *n* (%)	
	Parent	55 (65.5)
	Abortion	15 (17.9)
	Adoption	2 (2.4)
	Unsure	12 (14.3)

We identified self-reliance as a common and complex theme woven throughout women’s discussions about their pregnancies. When discussing their reactions, expectations, and decision-making about their pregnancies, approximately half of women (*n* = 40, 48%) spoke of self-reliance (specifically the need to rely on one’s own efforts and abilities), rather than those of others. Discussions of self-reliance overlapped with related discussions about prior pregnancy experiences, prior parenting experiences, current children, relationships, social support, decision making about the pregnancy, and maternal health. We found the theme of self-reliance to consist of several intersecting subthemes: 1) past experiences of self-reliance, 2) expectations of motherhood, 3) financial independence, 4) decision making about this pregnancy, and 5) self-reliance in parenting. Social support, or lack thereof, was a pervasive element of all subthemes, and was intimately related to women’s discussion of self-reliance.

### Past experiences of self-reliance

Many of our participants were already intimately familiar with the notion of self-reliance during pregnancy secondary to the absence of a partner or other social support in previous pregnancies or current experiences as mothers. For some, their previous experience with the reality of self-reliance may have led to decisions to parent, and for others the decision to terminate. For example, several women who were already mothers noted the following.

*I’m used to doing it by myself. I’m used to being the parent alone, not having to share, except for doctors’ appointments and delivery day. (Age 35)*

*I mean I* [parented other children] *by myself, and they’re doing good. (Age 38)*

Some participants noted both difficulty and gratification as parents who were already self-reliant. One woman who planned to continue her current pregnancy said:

*My daughter, her father’s not… in her life… as much as he should be... I’m doing everything, everything on my own. With schoolwork and parent teacher night, report card night, family support night, all of that. I mean I don’t mind … I love that she [will] always come to see me, the person that was there. (Age 26)*

Participants cited experiences raising children without social support and necessitating self-reliance as reasons why they believed parenting their expected children would be successful. Participants spoke of sacrifice and challenges in being self-reliant parents, but many also described feeling fulfilled by that role.

Similar descriptions were also offered by women planning abortion, perhaps related to their desire to care for and support the children they were already parenting. For example, one woman who planned to terminate (and did) expressed pride in her ability to be self-reliant for her young son:

*I do everything I can, for my son to have a good life. So I work…I basically do everything on my own for him… to see him in the morning wake up and smile and say ‘Mommy’, it’s just a good feeling. (Age 21)*

### Expectations of motherhood

Some participants took as a given that they would have to be self-reliant in both pregnancy and motherhood; for many women, self-reliance was a necessary element of both.

*You’re the mother… you have a mother and father but at the end of the day if it doesn’t work, you’re the mother. This is your child. So whether he is excited about it or not, I have to do what I have to do as a mom for my child. (Age 30)*

*He’s the man and I’m the woman. And at the end of the day, when you have a child, all the care for that child is based on the woman. (Age 37)*

Some participants described motherhood as a responsibility that required overcoming lack of social supports and embracing self-sacrifice in order to fulfill their duties as mothers.

*You’re having a baby, it’s going to be a struggle sometimes but you have to be able to provide and I’m not the type of person who, who just go and ask somebody, ‘hey can you, can you help me’ and stuff…I just, you know, feel like I would need to provide for my child. I don’t need nobody else to provide. (Age 30)*

For some women, the idea that the responsibility of parenting would ultimately (and sometimes inevitably) fall to them stemmed from a social norm that fathers are less duty-bound and reliable than mothers.

*And then at the end of the day, it’s mommy’s baby always. Like, he could get up and say whatever. Men can do whatever they wanna do, he’s not obligated to stay here whether we’re married, engaged, together or not. (Age 29)*

### Financial independence

Many women also referred to financial independence as a marker of self-reliance, and the reason why they were making the decision to parent, irrespective of their partners’ input on the matter.

*Yeah, I pay the high rent bill. He pays the cable and the gas and they don’t add up, so I got the say. This is how the world works! (Age 20)*

*I can make my own decisions. I work, I make my own money, pay my own bills, so my decision is my decision. If you’re not with it, don’t be around…. I don’t care. He probably wouldn’t be happy, he’d probably be a little discouraged, upset or something. But it’s my decision. (Age 21)*

Discussions about financial independence also overlapped with discussions about the influence of family on pregnancy decision-making and lack of social support from family, and sometimes shaped a participant’s plans to share (or not) the news of the pregnancy with others.

*[It’s] not that I don’t care what anyone has to say, but I don’t care what their opinions on it…if they have something negative to say I’m gonna say well…did you take care of any of my other kids? Would you like to pay a bill out of my house? Would you like me to write you a grocery list for us? … I don’t feel the urge to tell everyone cuz I’m like…this isn’t their baby. My household isn’t their household, I’ve been on my own since I was eighteen, I’ve lived in my own place, I’ve had my own car…if they find out, they find out. If they don’t I could care less. (Age 21)*

Conversely, several participants expressed that they did not see themselves as self-reliant because they lacked financial independence and stability. Some women voiced that they did not want to have to rely entirely on themselves in pregnancy or motherhood, which led some to question if continuing the pregnancy was the right decision. Several participants who felt this way also told researchers that they were planning abortion.

*I don’t want to be struggling…out here with two kids and then, you know, who knows? Me and my boyfriend only been together for a couple months… I’m not trying to do it by myself and I’m not trying to struggle and…I want to be more, I want to have a better job and stability. I don’t want to be living on food stamps….I’m just trying to be better, like better us, before having another kid. (Age 23)*

*And if I’m not stable myself, then I’m not gonna bring somebody into this world and have them struggle with me …. Stable, as um, financially having a roof over my head… mostly being prepared for it. I’m not at all. (Age 20)*

### Decision-making about this pregnancy

First, women displayed self-reliance simply in discussing decisions about their pregnancy. Many women expressed that they were relying solely on their own counsel to contemplate their decisions.

*Uh to be honest I could really care less what anyone else thinks because uh I’m 18. I’m gonna be 19 next month, and I mean, I’m an adult. I have to do what I have to do. I feel like [it’s] my decision. I mean they can’t really have no say, cause it’s my decision so. (Age 18)*

*I can do what I wanna do, I don’t have to be pressured into doing anything or listening to somebody. (Age 21)*

Furthermore, conceptualizations of absent or low social support and the need for self-reliance influenced the way some women approached making decisions about their pregnancies. Women cited self-reliance when considering whether or not to continue their pregnancies, including what it would mean to be single parents. For some participants, the knowledge that they would need to be self-reliant and even single-parents (either for the first time or again) influenced their plans to terminate, and for others this same knowledge appeared to factor into and reinforce their plans to parent.

Although a few women stated that their decisions depended in part on their partner’s wishes, more women expressed the sentiment that their partners’ opinions and roles were more or less irrelevant; in other words, they felt confident in their ability to be self-reliant and make decisions about continuing or terminating the pregnancy whether or not their partners stayed involved.

*But then I realized that I wanted this child no matter who the father is. So…I was like whatever, either you’re gonna be in our lives or not. It’s not gonna change anything, I’m gonna keep my baby. (Age 23)*

When asked how the father’s feelings about the pregnancy impacted her decision to parent, one participant said:

*No, it doesn’t influence me in any way cuz I’m a pretty strong-minded person…I don’t have to be with everyone, I can do my own thing. I don’t mind being alone. So it’s kinda like, whether he was OK with it or not, a baby is still gonna be here. (Age 21)*

The physical reality of pregnancy also shaped women’s perspectives on self-reliance and their pregnancy decision-making. Women saw their pregnancies as ultimately belonging to them, and so all decisions would be made accordingly. Two women who planned to parent expressed this sentiment:

*Men tend to be, you know, like (soft laugh), they don’t know. We’re the ones that carry [the pregnancy], that do all the work. (Age 24)*

### Self-reliance in parenting

Some women acknowledged that although a possibility, being self-reliant as a single mother without social support was not ideal. Many participants who planned either abortion or adoption pointed to the value of having a partner in parenthood.

*Right now I’m single, I don’t have anybody… you know I’m not ready for that (e.g. being a single mother) yet. (Age 21)*

*To be a good mother I think it takes a partnership. Of course single mothers do it, but I think a man and a woman should raise a child, not just a man or a woman. (Age 25)*

*I know a lot of families don’t stay together. But for me myself, to be able to provide for the child on my own…And if I’m not stable myself, then I’m not gonna bring somebody else into this world and have them struggle with me. (Age 20)*

Similarly, a few participants expressed that their previous experience as single parents influenced their strong preference for having partner support in the current pregnancy.

*I was by myself, had the baby by myself, took care of him by myself, until now… So, I just, kinda don’t want to go through that again, but I know that I’m with him now, that it might be different and that he might actually be there for me, but I don’t want to like have the baby thinking that. Oh, he’s there now and he’ll be with me and this will be a better pregnancy and stuff. (Age 25)*

One participant who planned to parent expressed that while her preference would be to have partner support, she was prepared to parent by herself if necessary.

*I see women do it all the time where you know they go through everything by their self… I just feel like… what mother doesn’t want a father there for her child?…And so I feel like that’s a big part for me. But I mean either way I’m going to do what I have to do. (Age 30)*

## Discussion

In this analysis of a racially and ethnically diverse urban population of women with new pregnancies, we identified self-reliance as a prevalent theme that emerged in discussions with women about how they felt the pregnancy would impact their lives, decisions, and relationships. Our findings suggest that both self-reliance and an awareness of the limits of self-reliance can have a substantial impact on a woman’s thoughts, feelings, and decision-making about a pregnancy. Experiences with and examination of self-reliance as it related to social support, previous pregnancies and experiences, expectations of motherhood, financial independence, decision making about the current pregnancy, and self-reliance in parenting, all contributed to a woman’s assessment of her new pregnancy.

Our findings advance understanding of self-reliance, especially as a potential response to lack of social support, in several ways. To our knowledge, this study is the first evaluation of women’s thoughts and expressions regarding self-reliance at the time of pregnancy diagnosis. We identified two previous studies that specifically report on self-reliance related to pregnancy [19, 20]. However, both studies were conducted in the postpartum period, and may be subject to recall bias. Ashaba et al. report on coping strategies used during pregnancy and childbirth by women living with HIV in Uganda (*n* = 20). They conducted postpartum qualitative interviews and identified self-reliance, mostly as it relates to financial independence and parenting, as one of five coping strategies these women used to navigate challenges during pregnancy and beyond [19]. In the second study we identified, Johansson et al. identified self-reliance as one of three main themes that emerged with first-time Swedish parents following same-day discharge from the hospital after childbirth (*n* = 21). In this study, the concept of self-reliance pertained to parents who needed to rely on their own instincts about newborn care at home, as opposed to asking for help or receiving assistance from healthcare professionals [20]. While helpful in defining some aspects of self-reliance and identifying it as an important theme among postpartum women, these two studies are limited to non-U.S. populations and are retrospective in nature. Our findings build on these smaller studies and clarify how self-reliance may function in a larger, urban, U.S. population of women in early pregnancy, prospectively contemplating pregnancy and parenting. We believe that characterizing self-reliance among pregnant women, often in the presence of limited or absent social support, is novel and an area that warrants further inquiry and analysis.

Strengths of our study include employing a qualitative approach using semi-structured interview questions, which allowed participants to express varied and at times contradictory emotions, thoughts, and feelings, which added complexity and richness to our data. The diverse racial and ethnic representation of our participants is also a strength, given prior research that has shown that the effects of social support and self-reliance vary across ethnic and cultural groups [24, 25]. Additionally, this study includes women in early pregnancy with varying pregnancy contexts (intention, wantedness, planning, timing, desirability, happiness) and outcomes (miscarriage, abortion, delivery), and therefore provides important perspectives not often captured in research about pregnancy. Our study may be limited by the lack of specific questions designed to evaluate self-reliance. Instead, the theme of self-reliance emerged from women’s discussions about their thoughts and feelings towards a new pregnancy. Another limitation of our study may be that our participants were recruited from a single geographic area; however, this region is diverse and generally representative of demographics in the United States [26].

Additional research is needed to explore self-reliance during pregnancy as there may be different and more complex sub-themes. It remains unclear whether self-reliance is a fixed character trait or rather a transient state of being that can be learned or cultivated over time. Future investigations into self-reliance in pregnancy could aid understanding of whether self-reliance is associated with a woman’s decision to continue or abort her pregnancy, if self-reliance can diminish the effects of low or absent social support, or if it positively or negatively affects different maternal and neonatal outcomes, such as postpartum depression or birthweight among women who decide to continue their pregnancy. Although there is evidence that interventions aiming to increase social support during the prenatal and postpartum period lead to better maternal and neonatal outcomes, findings are mixed [6, 27, 28]. Moreover, we do not know whether these same interventions would have any impact on women’s self-reliance, or if interventions aimed to increase self-reliance would lead to better outcomes as well, particularly in the absence of increased social support. Additionally, further evaluation regarding which types of social support and self-reliance effect these outcomes and for which ethnic and cultural communities, is warranted.

## Conclusion

Our findings suggest that self-reliance is an important aspect of women’s reproductive lives and choices. It’s a prevalent concept that is threaded through women’s thoughts about pregnancy, and may be an important coping strategy women employ to buffer the negative effects of diminished or absent social support. In the end, self-reliance may only take women so far in the absence of social support and financial resources. While healthcare providers can try to cultivate individual patient factors (self-reliance) that may be protective against negative maternal and neonatal outcomes, we must also consider the environment and supports that our healthcare systems and government provide for vulnerable women. As of 2015, 13% of all women aged 15–44 in the United States remain uninsured [29], and over 15 million women living below 250% of the federal poverty level are in need of publicly funded contraceptive services and supplies [30]. The current political climate poses further threats to family planning and preventive healthcare for underserved women [31–33], as well as to maternity and newborn care [34, 35]. Systems can either support or chip away at self-reliance, and in the face of shrinking benefits and worn safety nets, a woman’s self-reliance simply may be not enough.

## Additional file

Additional file 1: **Figure S2.** Individual and focus group interview guide. The interview guide with specific questions included that researchers used to structure participant interviews. (DOCX 120 kb)
