# Supplementary material for: “I’m used to doing it by myself”: exploring self-reliance in pregnancy
Source: BMC Pregnancy Childbirth. 2018 Oct 5;18:393. doi: 10.1186/s12884-018-2022-8 (PMC6173858; doi:10.1186/s12884-018-2022-8)
Supplement: Supplementary file 1 — Figure S2. Individual and focus group interview guide. The interview guide with specific questions included that researchers used to structure participant interviews. (DOCX 120 kb) [file 12884_2018_2022_MOESM1_ESM.docx]

**Figure 2. Individual and focus group interview guide**

*As you know we are doing a study to learn about women’s experience of pregnancy, especially about how they felt about being pregnant and the impact of the pregnancy on their lives. Some of the questions we will ask will seem rather personal and it is completely up to you to share what you are comfortable with and will be kept confidential to those in this room. You will never be identified by your real name.*

*If you decide you do not want to share your feelings that is fine.* ________________________________________________________________________ 1. When did you find out that you were pregnant?

2. Pregnancy can be intended, unintended or ambivalent. How would you describe your intention about this pregnancy?

3. Can you tell me your initial thoughts after receiving your positive pregnancy test?

a. How would you describe your initial feelings?

b. How would you describe your mood?

4. How are you feeling now?

a. How would you describe your mood?

b. Have your feelings changed at all since you received your pregnancy test?

c. What do you think caused this to change?

5. How do these feelings influence your decision about whether you want to parent, adopt out, or terminate the pregnancy?

6. What does it mean to you to be a good mother?

a. Do you think you’d be a good mother now? Why or why not?

7. Regarding the person you got pregnant with, how do you think he feels or would feel (if he doesn’t know yet) about your positive pregnancy test?

a. How do those feelings influence you?

b. How important is your relationship with him (whether in a relationship or not) to how you’re experiencing this pregnancy?

8. How has this pregnancy impacted your daily life?

a. How are things at home?

b. With finances?

c. With work/school?

d. How do you think they will they be impacted in the future? Positively or negatively?

9. How are your relationships with your friends and family lately?

a. How do you think your relationships will be impacted by this pregnancy in the future?

b. Will they change or stay the same? For the better or worse?

10. Who have you told about the pregnancy?

a. Who have you not told?

b. Why?

c. How have they responded to the news?

d. Were you pleased or displeased with their responses?

e. Were you surprised with their responses?

11. Are there additional issues related to your feelings and your pregnancy that you'd like to discuss?
